# Audible Noise Evaluation in Wind Turbines Through Artificial Intelligence Techniques

**DOI:** 10.3390/s25051492

**Published:** 2025-02-28

**Authors:** Mathaus Ferreira da Silva, Juliano Emir Nunes Masson, Murillo Ferreira dos Santos, William Rodrigues Silva, Iuri Wladimir Molina, Gabriel Miguel Castro Martins

**Affiliations:** 1 Robotictech Technology Services, Juiz de Fora 36036-230, Brazil; mathaus.silva@robotictech.com.br (M.F.d.S.); juliano.masson@robotictech.com.br (J.E.N.M.); williamrsilva97@gmail.com (W.R.S.); iurijunior940@gmail.com (I.W.M.); gabrielmcastro@outlook.com.br (G.M.C.M.); 2 Department of Electroelectronics, Federal Center of Technological Education of Minas Gerais (CEFET-MG), Leopoldina 36700-001, Brazil

**Keywords:** audible noise, artificial intelligence, wind turbine

## Abstract

In recent years, wind power has become a more attractive alternative energy source for overcoming environmental issues. Predictive maintenance is essential for wind power devices to ensure that these systems work reliably and with sufficient availability. This paper presents a method to work around failure detection in wind turbines using the sound emitted from their components. The proposed Artificial Intelligence (AI) model is based on unsupervised learning and image processing, through which the machine learning model learns to identify spectrograms from wind turbines under healthy conditions. The reconstruction of current data determines whether the input data have an uncommon noise, which translates into a possibility of failure or an effective one. The uncommon data are sent to a specialist network, which, through supervising learning, identifies a failure event and alerts operators to possible problems that the wind turbine could pass through, helping with preventive maintenance. The model offered satisfactory results in five tested wind turbines, in which some specific faults known by the operators were captured through the low similarity between the reconstructed data and the input. Additionally, this application could be extended to similar applications in industrial machinery within the scope of audible noises in rotative machine mechanisms.

## 1. Introduction

Wind power use is increasingly growing worldwide due to it being a clean energy, inexhaustible, and sustainable source, serving as a potential method to reduce the effects of global warming. In Brazil, wind power is the second most used power type, just behind hydroelectric power because of the vast territory and abundant rivers of the country [1]. The growth of wind energy usage worldwide was led by the United States of America, China, and India between the years 2016 and 2021 [2].

The wind power energy system is composed of wind turbines, the blades of which are rotated through wind kinetic energy and are coupled to a rotor with a specific gearbox and other mechanical and electronic components. Monitoring how these complex structures work together is still a challenge, especially regarding predictive maintenance, an essential technique that is required to prevent faults in wind turbines (their lifespan is around 20 years).

The emphasis on predictive maintenance arises from the fact that if a partial failure is not detected, a sequence of failures to the other components or even the entire wind turbine may occur, increasing the power outage time and, consequently, operational costs [3].

Gaining access to wind turbine mechanisms is also difficult because it involves high altitudes and requires specialized equipment and qualified personnel to execute maintenance tasks with security and precision [4]. This increases the importance of minimizing regular visits to the top of the structure by optimizing the maintenance processes and allowing a precise diagnosis.

Wind turbines eventually emit noises in the audible human range that can be perceived by people living around wind farms, and, in some cases, this may affect their health through symptoms such as insomnia [5,6]. Uncommon noises from the wind turbine nacelles, which are compartments that house the mechanical components of a wind turbine, can also be perceived by maintenance operators. Some of these noises can go unnoticed by the current supervisory systems used by most vibration sensors [7].

According to operators’ evaluations, singular noises that are unseen by the current supervisory systems are often correlated with failures and potential failures. Based on this, the development of a supervisory system that can capture those sounds from the wind turbines and generate alerts to the operators when the failure or pre-failure condition appears is necessary.

Building upon this idea, AI has emerged as a crucial tool for fault prediction, offering rapid responses, high accuracy, and reliable lead times before failures occur [8,9,10]. AI models can learn to detect abnormal conditions based on statistical patterns in the training data and continuously improve through experience [11]. Regarding wind turbine supervision (as with most rotational machines), monitoring systems commonly rely on vibration and acoustic emission sensors to detect failures, particularly in the gearbox. This critical component (housed within the nacelle) is highly susceptible to faults and is responsible for a significant amount of downtime in wind turbines. Previous studies on Swedish wind power plants reported that gearbox failures accounted for approximately 20% of downtime in such systems. More recent research indicates even higher rates (70% in small-scale and 50% in medium-scale turbines) [12,13]. Additionally, acoustic emission sensors can be installed on rotor blades to monitor structural conditions, such as fatigue, cracks, and increased surface roughness, by detecting abnormal vibrations [14].

Online monitoring has become the preferred approach for supervising sensors installed in the field due to their real-time detection and their capacity to integrate the sensor’s information into a Supervision, Control, and Data Acquisition (SCADA) system. This makes it possible to monitor the status of all turbines without being onsite. The SCADA system provides an interface and alarm system to alert the operator about each specific condition [15].

Non-Destructive Inspection Technologies (NDITs) play a critical role in ensuring low maintenance costs and extending the life cycle of structures. By enabling the detection of flaws and defects without causing damage, NDITs eliminate the need for operational interruptions, allowing structures to remain functional and efficient throughout the inspection process. This methodology involves non-invasive measurement techniques such as visual inspection, acoustic wave-based, optical, and imaging techniques, and electromagnetic fields. The machine learning and deep learning processes play important roles in NDITs (in data processing and evaluation) [16,17]. Image processing through acoustic emissions with a fundamental contribution by AI is explored throughout this work.

The use of data mining and analytics in industry has grown in recent years through the development of eight unsupervised learning and ten supervised learning algorithms, which compose the state-of-the-art methods. Regarding unsupervised learning methods, unlabeled data require K-means clustering, kernel density estimation, self-organizing maps, Gaussian mixture models, manifold learning, and support vector data descriptions. On the other hand, supervised learning, which is useful for fault classification and identification, has been applied in methodologies such as Principal Component Analysis (PCA), partial least squares, Fisher discriminant analysis, multivariate linear regression, neural networks, Support Vector Machine (SVM), nearest neighbor, Gaussian process regression, decision tree, and random forest [18].

The use of AI in wind turbine predictions detailed in Rashid et al. [19] involved the application of machine learning methods in output power prediction. Data collection involved SCADA parameters (such as the wind direction, wind speed, and output temperature) and was conducted over a period of two years to collect information on all of the data variance to train, recognize patterns, and predict the output power.

In terms of fault diagnosis, Carratù et al. [20] presented unsupervised anomaly detection methodologies for industrial processes involving a machine learning application. They emphasized data clustering, a trend used in machine learning methods to group objects with similar features. The results showed the possibility (through methodologies such as K-means clustering) of grouping normal and abnormal conditions, even for unbalanced datasets. The present methodology (which joins K-means clustering and classical frequency analysis such as Short-Time Fourier Transform (STFT) used in spectrograms) eliminates the true positives, increasing the precision level compared to the adaptive model and deep auto-encoder. This could be used for pre-failure detection in wind turbines, whereby, after a generalist model detects the presence of a presumable fault condition, the clustering of data separates the known faults.

Natili et al. [21] discussed the application of support vector data to predict the output data of wind turbine bearings with the SCADA features temperature and oil pressure using Support Vector Regression (SVR). These features were used to provide preliminary advice for previously faulty conditions that were known. After that, a vibration analysis in the time domain involving accelerometers placed on the gearbox casing was conducted, in which the data were separated using PCA. This method was shown to be a very powerful tool for early fault diagnosis, allowing the discovery of the fault around 5 months before the turbine’s stop-point. Through temperature analysis, a faulty drift could be identified 4 months before the turbine stopped. From the PCA, more than 70% of the chunk points were identified correctly as showing damaged conditions. This methodology could also be used in data clustering to classify anomalies such as pca usage. In contrast, instead of using SCADA parameters, in this work, our model uses sound as the primordial and complete source of fault diagnosis. SCADA parameters are used as assistants to build datasets by classifying the different conditions related to the behavior of each metric.

Another interesting study conducted by An and Cho [22] demonstrated the use of a variational auto-encoder for anomaly detection, which is the field explored in this paper. Here, the training process is conducted with data that represent normal conditions. The idea is to assess the reconstruction quality of images: if an image is well reconstructed, the data should be similar to the training dataset; if not, this may indicate a possible fault condition. The results underscore the improved performance compared with the auto-encoder and PCA and the possibility of using the data reconstruction to analyze the underlying causes of anomalies.

As mentioned previously, images are the most representative data that are used in deep and machine learning models. Such methods involve the building of a dataset to teach the model, pre-processing to eliminate undesirable noises, segmentation to analyze each part of the image, and finally, feature extraction [23,24,25].

The work conducted by Zhang et al. [26] introduced a supervision method based on the combination of the input and reconstructed images to inspect the tinfoil fault inline production. Comparison metrics such as the Structural Similarity Index Measure (SSIM) and Peak Signal-to-Noise Ratio (PSNR) were used to rate the tinfoil condition using the reconstruction quality. At the training step, the model only learned the normal areas of the tinfoil. Then, once it knew the normal conditions, the model could identify abnormalities without needing to label them. The results of this robust auto-encoder model showed an accuracy that was 4% higher than that of traditional methods, which ensured the ability of this unsupervised learning method to capture small details in tinfoil. This supervising methodology contributed to the reading of spectrograms at specific moments and, through teaching the model to recognize healthy conditions, the auto-encoder could conduct the reconstruction. The correlation between the output and input produced a metric that indicated the reconstruction quality and therefore, the probability of a fault being present.

Regarding audio classification, spectrograms are also commonly used. They are time–frequency representations of audio signals. Thus, the audio classification follows the same stages as the image processing mentioned [27]. The method developed by Zhang et al. [28] extracted features from audio spectrograms using a Convolutional Neural Network (CNN) to conduct event recognition. The robustness of using a CNN in the spectrogram image feature to allow sound event recognition was shown to have an excellent mean classification accuracy of around 94% under four different signal noise ratio conditions (clean, 20 dB, 10 dB, and 0 dB). The CNN application could incorporate the auto-encoder structure to improve the performance of reconstructions. The CNN model could also be used in speech recognition algorithms and some specific sound detection applications to filter undesired sounds in the pre-processing step due to the processing power required to reach deep layers and separate specific sounds.

Separating different audio sources is still a big challenge in deep learning, especially regarding the generalization of unseen sounds. This task is very useful in many areas, especially in music, where the audio separation from different instruments is present. Chen et al. [29] introduced a methodology to separate audio sources through the zero-shot technique with weakly labeled data. This methodology was shown to outperform state-of-the-art methods through having higher SDR metrics than the other known methods. It is also an interesting method to apply in the pre-processing step, as it can be used to separate recorded audio from undesired noise, such as fans which are used to control the temperature, tools, and speech produced by the operators working inside the structure of a wind turbine.

Based on the information presented, anticipating failures plays a crucial role in the optimization of maintenance planning, particularly in wind turbine operations, where unexpected downtime can lead to significant financial losses. By enabling early fault detection, the proposed model supports predictive maintenance strategies, allowing operators to schedule repairs before critical failures occur. This proactive approach minimizes unplanned interruptions, extends the lifespan of components, and improves the overall system efficiency. In contrast to traditional reactive maintenance methods, which rely on fault occurrence, predictive maintenance reduces operational costs and enhances the reliability of wind energy generation.

Within this context, this paper introduces a novel methodology for wind turbine fault diagnosis in which the noise emitted by mechanical components is analyzed. The approach involves sound capture using commercially available devices, such as microphones and microprocessors encapsulated within the nacelle. Once recorded, the audio goes through a processing pipeline in which spectrogram segments are analyzed. If a potential fault is detected, the data are directed to dedicated models for classification.

This non-invasive and cost-effective solution offers an alternative to conventional supervisory systems, which rely on multiple sensors attached to various components inside the wind turbine. In contrast, this approach utilizes a single microphone to monitor all nacelle components simultaneously. Given this scenario, the paper explores this underutilized methodology for fault diagnosis in rotating machinery, leveraging audio processing as the primary technique for feature extraction within the wind turbine nacelle.

By integrating machine learning techniques, such as auto-encoders, the proposed approach enhances automation in addition to providing the high performance and predictive efficiency benefits demonstrated in related studies. Additionally, this research emphasizes the role of image processing, employing a unique strategy that uses various audio features to train a machine learning model. The model reconstructs the input data and, by evaluating similarity metrics, compares the output with the original data to classify audio segments as either normal or abnormal.

The paper is structured as follows: Section 2 presents an overview of the machine learning models used to identify the anomaly conditions and the structure of the models used in this application; Section 3 explores the hardware construction of the audio recording device; Section 4 details the steps taken to process the audio using neural network models and discusses the specialist models designed to label the faults; Section 5 shows the experimental results collected from wind turbines in the operation regime and demonstrates fault detection and event characterization; and Section 6 discusses and concludes the paper, as well as presents opportunities for future work.

## 2. Proposed General Model Structure

To evaluate whether data are from healthy or non-healthy conditions, it is necessary to know what the data characteristics in each scenario are. Since wind turbines generally work normally, it is difficult to build a failure dataset for supervising learning due to the insufficient quantity of data available to train a machine learning model.

From this emerged the unsupervised learning method based on auto-encoders, which uses only healthy data for learning and replicates these in the output. The data that differ from the trained dataset produce a bad reconstruction, indicating a possible non-healthy condition. Furthermore, auto-encoders are an unsupervised method that uses an approximation to reproduce the input data that pass through compression in hidden layers, extract important features, and return them to the decoder to predict the original image. For this project, two distinct auto-encoder models were used in parallel to enhance reliability.

The average loss function of an auto-encoder for *N* input training data follows Equation (1), where the difference between the input data $x_{i}$ and the reconstructed data is given by the decoded layer, which is represented by the function $f_{\phi }$, and this is converted back to the original dimensions through the encoder layer represented by $g_{\theta }$ [30]:

(1) $$\mathrm{LAE}\left(\phi ,\theta \right)=\frac{1}{N}\sum_{i=1}^{N}L\left(x^{\left(i\right)}-g_{\theta }\left(f_{\phi }\left(x^{\left(i\right)}\right)\right)\right).$$

### 2.1. Denoising Auto-Encoder

A Denoising Auto-Encoder (DAE) is a stochastic extension of an auto-encoder that is trained to learn how to reconstruct data by receiving corrupted data in the input and return clean data that is similar to the original version [31].

**Figure 1:**
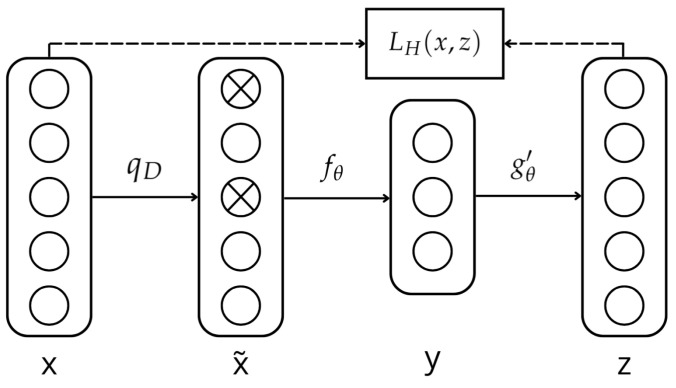
DAE representation.

**Figure 2:**
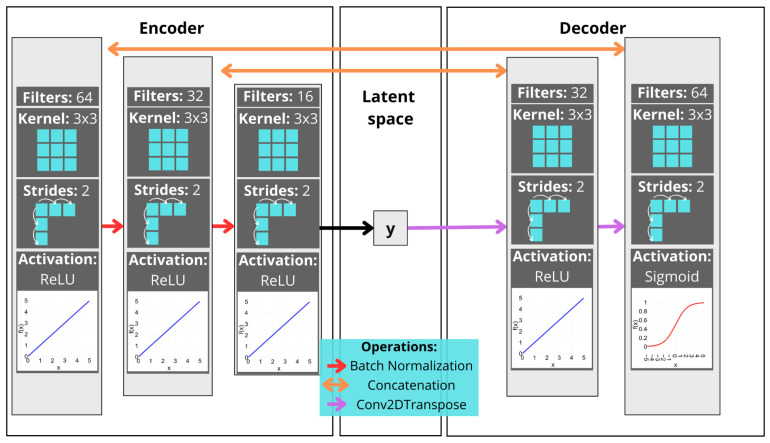
DAE architecture.

Figure 1 demonstrates this principle. A data point *x* is corrupted to $\tilde{x}$. The auto-encoder maps it to the hidden representation $y=f_{\theta }\left(\tilde{x}\right)=s\left(W\tilde{x}+b\right)$ and reconstructs it to $z=g_{\theta }^{\prime }\left(y\right)=s\left(W^{\prime }y+b^{\prime }\right)$. *W* and *b* are the weight and bias matrices, respectively. Thus, the parameters are trained to minimize the average reconstruction error given by $L_{H}\left(x,z\right)=H\left(Bx\|Bz\right)$, which is typically the Mean Squared Error (MSE) and approximate *z* of the original input *x*:

This forms a convolutional auto-encoder model that is able to deal with spectrogram image processing, as shown in Figure 2:

In the encoder layer, the data are compressed by passing through the batch normalization process in hidden layers until maximum compression is achieved. This process is responsible for accelerating the training of deep learning models, making the neural networks more stable by protecting against outlier weights, enabling higher learning rates, and reducing overfitting [32].

As demonstrated by Equation (2), the normalization of an input data point *x* in the *k*th position is the difference between the input data and the mean ($\mu_{\mathrm{batch}}$) of the data points within the current batch, which consists of *m* samples (batch-size) divided by the standard deviation of the batch $\sqrt{\sigma_{batch}^{2}}$:

(2) $${\hat{x}}^{\left(k\right)}=\frac{x^{\left(k\right)}-\mu_{\mathrm{batch}}}{\sqrt{\sigma_{\mathrm{batch}}^{2}+\epsilon }}=\frac{x^{\left(k\right)}-\frac{1}{m}\sum_{i=1}^{m}x_{i}^{\left(k\right)}}{\sqrt{\frac{1}{m}\sum_{i=1}^{m}{\left(x_{i}^{\left(k\right)}-\mu_{\mathrm{batch}}\right)}^{2}+\epsilon }},$$

where a small term ϵ is added to the denominator to prevent division by zero.

Applying a scale parameter γ and the bias β parameter to the normalized value ${\hat{x}}^{\left(k\right)}$ gives the equation of batch normalization $BN_{\gamma ,\beta (x_{i})}$:

(3) $$BN_{\gamma ,\beta (x_{i})}=y^{\left(k\right)}=\gamma {\hat{x}}^{\left(k\right)}+\beta .$$

The 64 filters (each one with a kernel of 3 × 3 size) slide across the entire image matrix, extracting key features such as textures, corners, and edges. The Rectifier Linear Unit (ReLU) activation nullifies negative activation. As indicated by Equation (4), the application of a ReLU in a neuron *x* improves the learning speed in the hidden layers, reducing the computational cost. This was demonstrated by Glorot et al. [33]. The strides define the number of neurons skipped, aiming to accelerate the training process but decrease the recognition accuracy [34]:

(4) $$\mathrm{ReLU}\left(x\right)=\max\left(0,x\right)=\left\{\begin{array}{cc} x & \mathrm{if}\;x>0\\ 0 & \mathrm{if}\;x\leq 0 \end{array}\right..$$

The latent space is the compact representation of the data, which is improved at each training step as the model learns to capture the most important aspects of the original data in general in a deterministic form, in the case of a DAE.

In the decoder layer, the data are reconstructed from the compressed representation of the last encoder hidden layer. From this layer, the data pass through the transpose process, which is the upsampling process that converts the data back to the resolution of the original spectrogram. The ReLU function is also applied to the hidden layer and, in the output, the sigmoid function is used, as presented in Equation (5):

(5) $$\sigma \left(x\right)=\frac{1}{1+e^{-x}}.$$

This function represents any real value in the interval between 0 and 1, which is required in the image normalization process. This function is commonly used to adapt the output neurons to this range.

**Figure 3:**
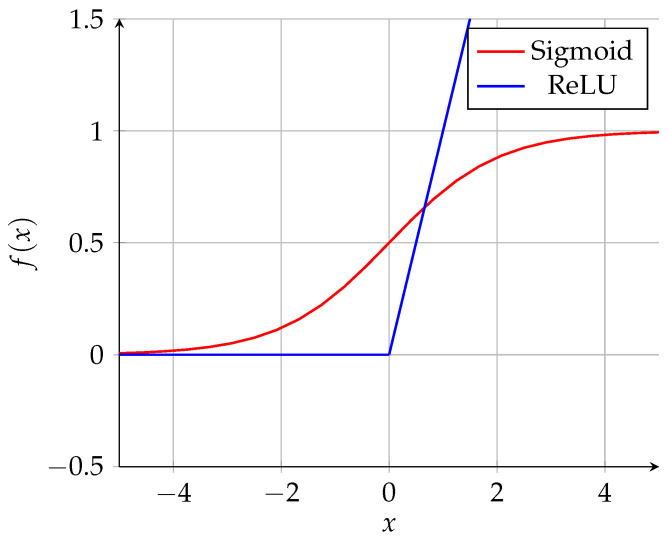
ReLU and sigmoid activation functions.

Graphically, the ReLU and sigmoid functions used in Equations (4) and (5), respectively, are both represented in Figure 3:

The concatenation process, commonly applied in DenseNet [35], allows the model to receive the output of previous layers, improving the information flow but increasing the computational cost. The *L*th layer carries the feature map of all previous layers $x_{0},x_{1},\ldots ,x_{n}$, which is used as the input:

(6) $$x_{L}^{\prime }=H_{L}^{\prime }\left(\left[x_{0},x_{1},\ldots ,x_{\ell -1}\right]\right),$$

through the application of a non-linear transformation $H^{\prime }$ using a composition function that involves batch normalization, ReLU, or pooling.

### 2.2. Variational Auto-Encoder

A Variational Auto-Encoder (VAE) is a probabilistic version of the traditional auto-encoder. Its loss function, presented in Equation (7), combines two main terms: the data reconstruction error (data consistency) and a regularization term that forces the latent distribution to approximate a prior distribution (regularization):

(7) $$L\left(\theta ,\phi ;x\right)=E_{q_{\phi }\left(z|x\right)}\left[\log p_{\theta }\left(x|z\right)\right]-D_{KL}\left(q_{\phi }\left(z|x\right)\;∥\;p\left(z\right)\right).$$

Its main distinguishing feature is that it learns to represent data in the latent space *z* as probability distributions using the Gaussian function $p_{\theta }\left(z\right)=N\left(z;0,I\right)$ and the conditional probabilities parametrized by the encoder $p_{\theta }\left(x|z\right)$ and the decoder $q_{\phi }\left(z|x\right)$, instead of using fixed points. This approach allows it to generate new samples that are similar to the original ones by sampling from this latent distribution [36].

The expectation $E_{q_{\phi }\left(z|x\right)}$ is the expected average value, given by the integral of the probability density function ($E\left[X\right]=\int_{-\infty }^{\infty }xp\left(x\right)\;dx$). The Kullback–Leibler (KL) divergence from the term $D_{KL}\left(q_{\phi }\left(z|x\right)\;∥\;p_{\theta }\left(z\right)\right)$ of the equation allows the distance between the two probability distributions to be measured $p_{\theta }\left(z\right)$ and $q_{\phi }\left(z|x\right)$. This is expressed by Equation (8):

(8) $$D_{KL}\left(q_{\phi }\left(z|x\right)∥p_{\theta }\left(z\right)\right)=\int q_{\phi }\left(z|x\right)\log\frac{q_{\phi }\left(z|x\right)\left(z|x\right)}{p_{\theta }\left(z\right)}\;dz.$$

**Figure 4:**
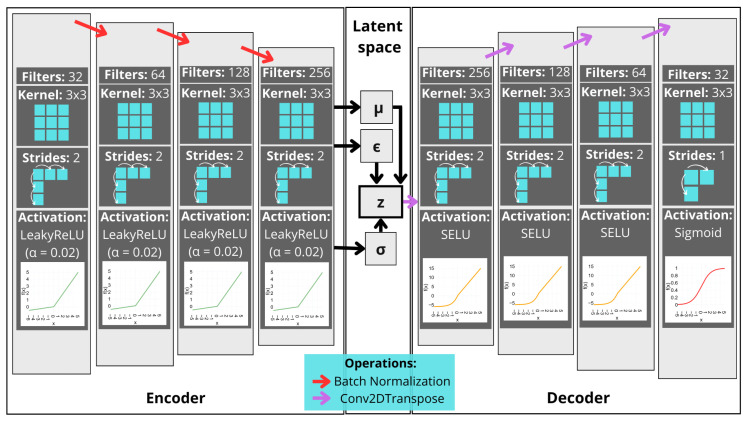
VAE architecture.

The VAE model used in this paper is presented in Figure 4:

The model’s encoder incorporates additional layers and uses the LeakyReLU activation function as its primary activation function. A LeakyReLU is an enhanced version of the traditional ReLU function. While a ReLU outputs zero for any negative input, a LeakyReLU allows a small, non-zero slope for negative values, helping to mitigate the issue of “dying neurons”, which is often encountered with ReLUs.

Furthermore, the slight negative slope ensures that some gradient is still present for negative activation. This may be crucial for maintaining the model’s stability during training and promoting better convergence in deep neural networks.

**Figure 5:**
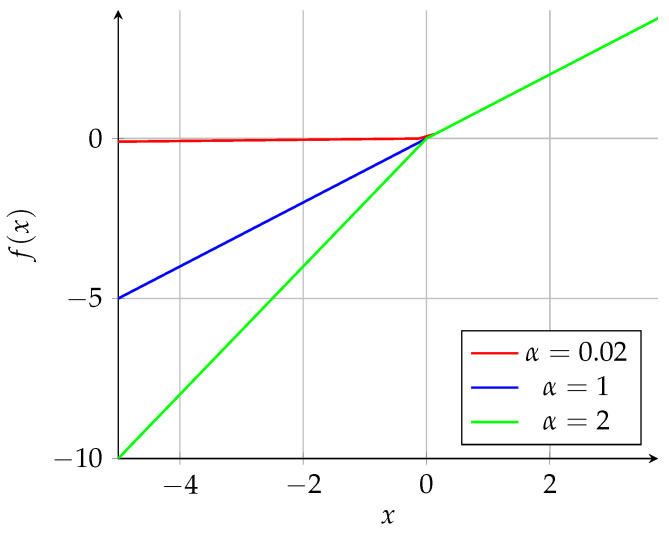
LeakyReLU activation function with different α parameters.

Equation (9) represents the LeakyReLU operation. Figure 5 provides a graphical representation under different conditions. The sigmoid function was applied to the output, as performed with a DAE [37]:

(9) $$f\left(x\right)=\left\{\begin{array}{cc} x & \mathrm{if}\;x>0\\ \alpha x & \mathrm{if}\;x\leq 0 \end{array}\right..$$

For α=0, the LeakyReLU turns into the ReLU, while for other values, the line equation is expressed with a non-zero coefficient α. A subtle value of α=0 is applied in the VAE encoder layer, as shown in Figure 4.

In the decoder layer, the Scaled Exponential Linear Unit (SELU) function proposed by Klambauer et al. [38] is used. This is defined by Equation (10), which also prevents the gradient from weakening or vanishing during the training process:

(10) $$f\left(x\right)=\lambda \left\{\begin{array}{cc} x & \mathrm{if}\;x>0\\ \alpha (e^{x}-1) & \mathrm{if}\;x\leq 0 \end{array}\right.,$$

where λ is a scale factor (by default, it is set to approximately 1.0507); the angular coefficient α is around 1.67326 [39].

**Figure 6:**
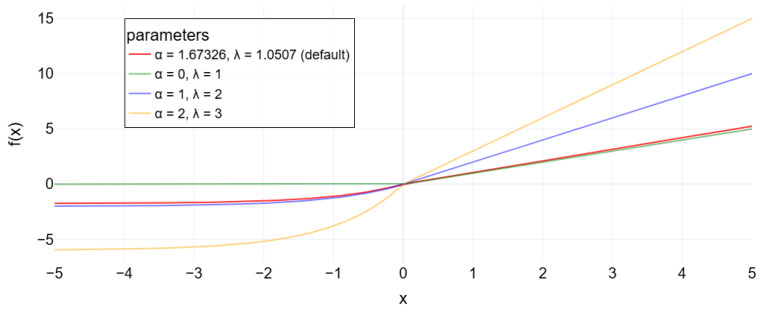
SELU activation function with different α parameters.

Figure 6 indicates the activation function behavior through the values of the neurons represented in the x-axis:

## 3. Audio Processing

To capture the noises emitted near the gearbox, a microphone was placed inside the nacelle to record the surrounding environment. The audio must be sent to a dedicated device, such as a computer, to be saved. As long as this computer is dedicated to this specific task, a large available storage capacity is not necessary. Making the records available is also crucial so that they can be obtained and passed through treatments.

**Figure 7:**
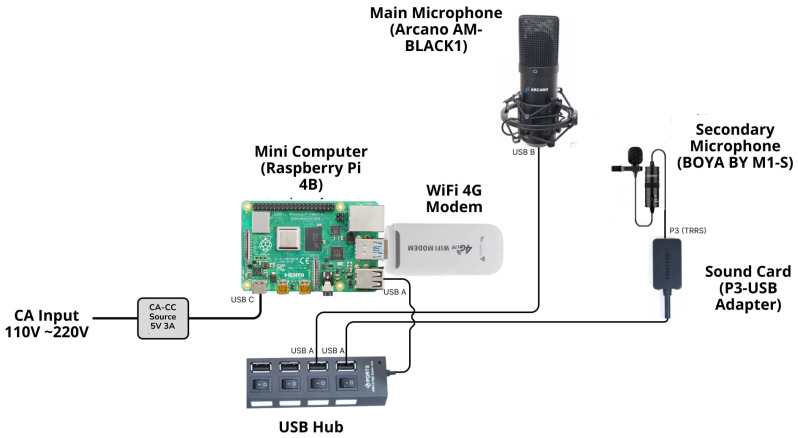
Schematic of the audio recorder device.

Considering those requirements, a dedicated hardware device was assembled composed of commercial components to capture audio and store it in an Amazon AWS cloud server. Figure 7 shows the hardware assembly schematic, followed by the details of the respective items.

The description of each component is presented below:

- CA-CC source: converts the CA from the distribution to provide the minicomputer with adequate voltage, providing 5V 3A in AC-DC.
- Minicomputer: The microprocessor Raspberry Pi 4B, which is responsible for processing the audio record routines, is installed inside the structure. This device stores the logic processes and, through its peripherals, allows connections with other devices such as the modem 4G and the USB hub. In addition to its portability, this device is widely recognized for its low cost, strong community support, and adequate performance.
- Wi-Fi 4G modem: allows the Raspberry Pi to be connected to the internet to save audio in the AWS cloud to be accessed.
- USB hub: multiplies the USB inputs of the Raspberry Pi, allowing it to connect to more than one microphone and ensuring that there is sufficient quality and minimum interference in the recording routine.
- Main microphone: An external microphone that is used to capture sound from the wind turbine components. It is flexible due to the length of the cable, which allows it to be positioned close to the wind turbine’s noisy components. The ARCANO AM-BLACK-1 model was chosen due to its high sensitivity compared with other models tested.
- Secondary microphone: Provides support/redundancy to the main microphone. It allows audio to be captured under adverse conditions, thanks to its lapel microphone design and its internal placement within the compartment. The microphone model BOYA BY M1-S contains a P3 output that is adapted to connect via USB.
- Sound card: this adapter converts the P3 output from the secondary microphone to a USB output to connect it to the USB hub.

**Figure 8:**
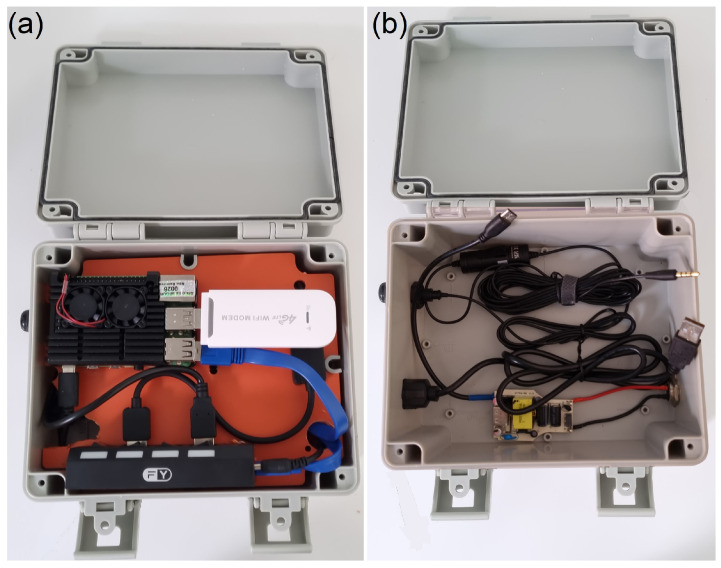
Audio recorder device top view: (**a**) With device support, showing the components. (**b**) Without device support showing, where the cables are hidden.

Figure 8 illustrates the devices enclosed in a protective box to shield them from dust and humidity. The prototype is divided into two compartments: the top houses the previously mentioned components, while the bottom contains the coiled cables:

Each device was placed in a single wind turbine nacelle. The device captured sound from the principal components, such as the gearbox and bearings, which are the most troublesome systems, as mentioned by Joosse et al. [14].

**Figure 9:**
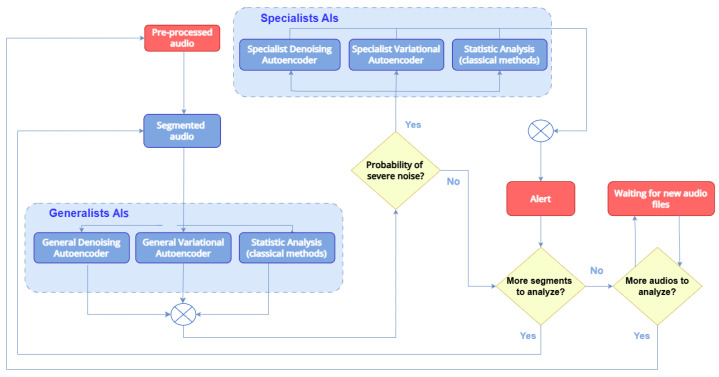
Audio processing flowchart.

The audio processing routine followed the flowchart presented in Figure 9, which shows, in a macro view, the fundamental tasks from the point of audio capture until the output to the supervisory system:

After the Raspberry Pi concluded the audio recording tasks, the audio was stored on the AWS server to be captured by the dedicated API. The AWS cloud service allowed the stored MP3 or MPEG files to be downloaded and processed. Five-minute audio files for each hardware type were stored in a bucket with the epoch format matching the start record time.

During the audio pre-processing stage, several parameters were considered to enhance the audio quality and optimize information capture. These included general audio parameters such as the hop length, Nonuniform Fast Fourier Transform (NFFT), and decibel range, and image-related features such as the spectrogram dimensions and color-map selection.

To conduct a time-domain analysis of the entire audio behavior, it was necessary to segment each audio type to obtain a short representation and extract the metric from that short period.

After audio segmentation was conducted to capture the wind turbine conditions in each time interval, the spectrograms generated passed through generalist AI models. They were responsible for identifying possible failures in wind turbines. Models based on deep convolutional auto-encoders classified the quality of the spectrogram reconstruction (using similarity metrics between the output and the input of the model).

If the model considered the fault possible, the spectrogram was sent to the specialist AI blocks to label the fault condition. Through the knowledge of the faults due to experience and clustering techniques presented in related works, it was possible to separate the conditions using thresholds. In the following sections, we explain the generalist and specialist AIs structures and show how the classic methods help with this purpose.

## 4. Proposed Detection Method

This section presents the steps developed in this work for fault detection in wind turbines using auto-encoder models, as presented in Section 2. Then, the methodology is explained and divided into training, an inference stage, and an evaluation of the wind turbine’s health. Lastly, the methodology adopted to specify the fault is presented.

### 4.1. Data Processing

To build both models, Tensorflow, a complete open-source library released by Google, was used to deal with the machine learning process.

**Figure 10:**
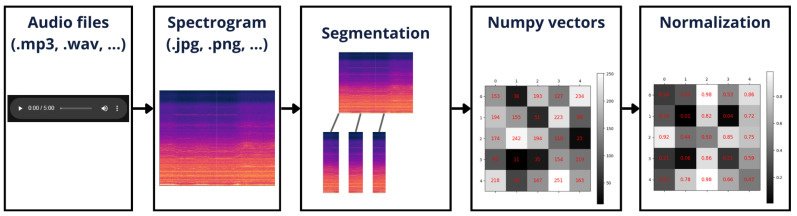
Data processing to feed the auto-encoders.

To process audio data effectively, it was necessary to follow the steps illustrated in Figure 10:

This figure demonstrates the workflow used to prepare and interpret audio data. It starts with the raw audio, passes through the spectrogram images, and finally, transforms it and normalizes it to a matrix that is suitable for the input of the auto-encoders.

Firstly, the audio of the wind turbines is stored in the AWS bucket and saved under default codification formats, such as MP3 and WAV. For image processing, the audio files are converted to spectrograms, and the spectrograms are segmented to allow a detailed analysis at each moment.

To enable models to process images, they must be represented as matrices, where each position corresponds to a pixel containing the numerical value of its color. For RGB images, this is represented as a three-dimensional matrix with dimensions for width, height, and three color channels. Each channel at a specific pixel location holds a value that indicates the intensity of the color. However, grayscale images can be more computationally efficient, since they process three times fewer pixel values than RGB, often delivering comparable results for many applications.

In digital images, the pixel brightness is typically represented using values within the range of 0 to 255, where 0 corresponds to the darkest shade (black) and 255 represents the brightest shade (white). This range is commonly used for raw image data to define the intensity level of each pixel. However, deep learning frameworks such as TensorFlow 2.10.0, along with most neural network models, are designed to operate more efficiently with normalized data values of between 0 and 1. This normalization process involves the scaling of pixel intensity values so that they fall within this smaller range, which not only improves the computational stability but also ensures that the model can correctly interpret and process the input data. Proper normalization is a critical pre-processing step, as it aligns the data representation with the expectations of the underlying algorithms, enabling more effective training and inference.

### 4.2. Training Stage

To effectively train the convolutional auto-encoders, it is essential to construct a robust dataset of spectrogram images derived from audio recordings of wind turbines operating under normal conditions. This allows the model to learn patterns associated with healthy turbine behavior and accurately reconstruct them with high reliability.

**Figure 11:**
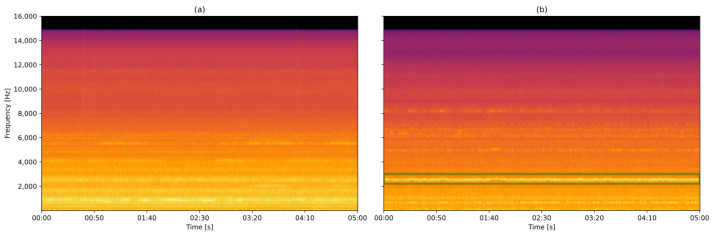
Audio of a wind turbine under (**a**) weak and (**b**) strong wind conditions.

A key challenge in this process is gathering spectrograms that encompass a diverse range of scenarios while still representing a properly functioning wind turbine. Variations in these images arise from factors such as wind speed, as illustrated in Figure 11. The software Audacity 3.4.2 was used to read spectrograms:

The braking system, commanded by the Doubly Fed Induction Generator (DFIG), is activated when the generator reaches a specific speed, with the aim being to control the generation [40]. This behavior is noticeable at frequencies of around 700 Hz (marked in green), which, at first impression, could sound noisy, but they represent the normal operation of the wind turbines, which the model needs to learn.

The other sound that is often emitted from the inside of the nacelle is the fan, which is used for cooling under high temperatures. This sound is characterized by a foggy appearance in the spectrogram and can be misidentified as a non-healthy condition.

Thus, a dataset with different speed conditions was produced with the help of a SCADA system. It classified speed levels, as presented in Table 1, aiming to provide a balanced dataset that could be used to train the DAE and VAE models:

Therefore, hyperparameters are correlated with the model’s performance, and tuning them is crucial for obtaining high machine learning model performance. The primary hyperparameters configured in the models were the number of epochs, the batch size, and the learning rate. Epochs represent the number of complete passes through the entire dataset during training. The batch size refers to the number of samples processed simultaneously, with model weights updated after each batch. Finally, the learning rate determines the step size used for adjusting the weights during training [41].

**Figure 12:**
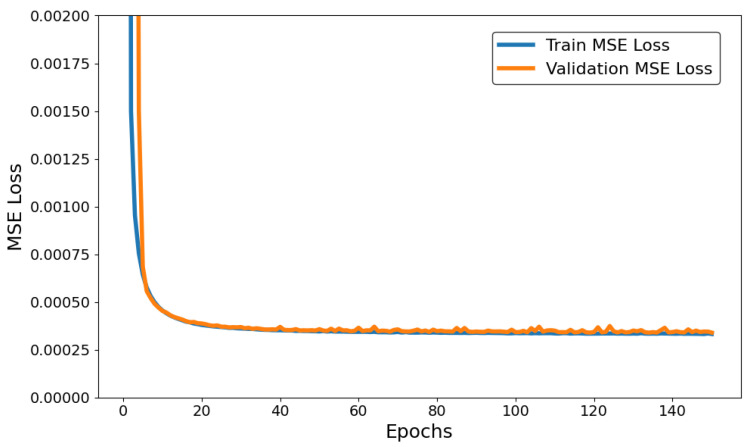
DAE performance over 150 epochs.

**Figure 13:**
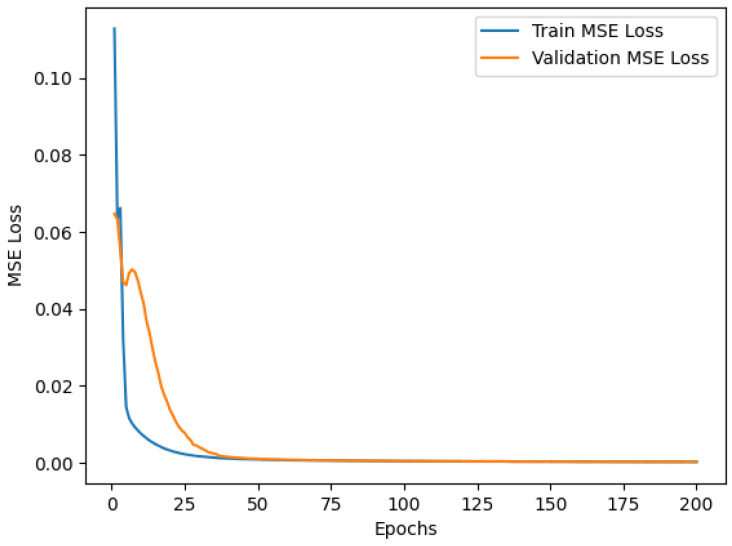
VAE performance over 200 epochs.

During training, the data were divided into two sets to prevent overfitting: training and validation. The training set adjusted the model’s weights, while the validation set was used to evaluate the performance and fine-tune hyperparameters. The goal was to ensure that the model generalized well to new data, avoiding both over- and underfitting. Figure 12 and Figure 13 present the model’s performance during the training process, presenting low MSE losses for the training and validation groups throughout the epochs. It ensured the ability of both auto-encoders to reconstruct the normal data:

### 4.3. Inference and Evaluation

To evaluate the healthy conditions, each spectrogram reconstruction of both auto-encoders was compared with the respective inputs and by using metrics to compare the images, a similarity level was associated with the quality of the reconstruction and consequently, the similarity of the current spectrogram to normal conditions was determined.

**Figure 14:**
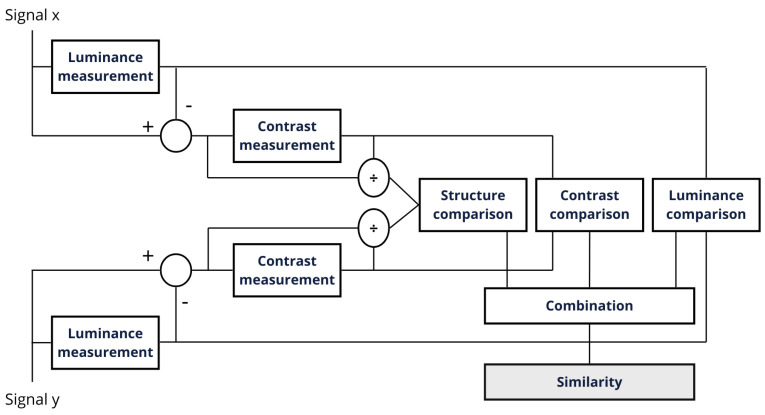
SSIM system.

The first metric used to evaluate this reconstruction quality was the SSIM. This metric, developed by Wang et al. [42], analyzes the image features luminance, structure, and contrast to calculate the similarity between two images. Figure 14 demonstrates how the SSIM is calculated:

The luminance is defined as the combination of the light intensity with the reflection properties. Equation (11) describes this attribute in terms of the average of the *N* signals from each non-negative image signal *x*. Then, the luminance comparison l(x,y) is defined as a function of $\mu_{x}$ and $\mu_{y}$:

(11) $$\mu_{x}=\frac{1}{N}\sum_{i=1}^{M}x_{i}.$$

As the term implies, it is the perceived difference between the darkest and lightest parts of an image. It can be approximated to a standard deviation from the signals of the image (σ), as described in Equation (12). The contrast comparison from both images c(x,y) (as follows in the diagram) is a function of $\sigma_{x}$ and $\sigma_{y}$:

(12) $$\sigma_{x}=\sqrt{\frac{1}{N-1}\sum_{i=1}^{M}{\left(x_{i}-\mu_{x}\right)}^{2}}.$$

After this, the normalization process given by the block structure comparison is achieved using Equation (13). The longer it takes, the closer it is to the average value and the lower its variance:

(13) $$s_{x}=\frac{x-\mu_{x}}{\sigma_{x}}.$$

Finally, the combination block is expressed by Equation (14). This combines the three attributes mentioned:

(14) $$S_{x}=f\left(l\left(x,y\right),c\left(x,y\right),s\left(x,y\right)\right).$$

where the luminance l(x,y), contrast c(x,y), and structure comparisons s(x,y) are represented by Equations (15), (16), and (17), respectively:

(15) $$\begin{array}{ccc} l(x,y) & = & \frac{2\mu_{x}\mu_{y}+C_{1}}{\mu_{x}^{2}+\mu_{y}^{2}+C_{1}}, \end{array}$$

(16) $$\begin{array}{ccc} c(x,y) & = & \frac{2\sigma_{x}\sigma_{y}+C_{2}}{\sigma_{x}^{2}+\sigma_{y}^{2}+C_{2}}, \end{array}$$

(17) $$\begin{array}{ccc} s(x,y) & = & \frac{\sigma_{xy}+C_{3}}{\sigma_{x}\sigma_{y}+C_{3}}, \end{array}$$

and $C_{1}=C_{2}={\left(k_{2}L\right)}^{2}$ and $C_{3}=C_{2}/2$.

The SSIM is:

(18) $$\begin{array}{ccc} \mathrm{SSIM}(x,y) & = & {\left[l\left(x,y\right)\right]}^{\alpha }{\left[c\left(x,y\right)\right]}^{\beta }{\left[s\left(x,y\right)\right]}^{\gamma },\; \end{array}$$

(19) $$\begin{array}{ccc} & = & \frac{\left(2\mu_{x}\mu_{y}+C_{1}\right)\left(2\sigma_{xy}+C_{2}\right)}{\left(\mu_{x}^{2}+\mu_{y}^{2}+C_{1}\right)\left(\sigma_{x}^{2}+\sigma_{y}^{2}+C_{2}\right)}, \end{array}$$

where the parameters α, β, and γ are useful to emphasize the relative importance of each parameter. In this work, these were simplified to α=β=γ=1.

The PSNR was another metric used as the output of the models, which is commonly employed as a quantitative measure of the reconstruction quality in the image compression field. This metric has a correlation with the MSE, as shown in Equation (20). It is ten times the logarithmic function of the difference between the maximum pixel value squared and the MSE:

(20) $$\mathrm{PSNR}=10\log_{10}\left(\frac{{\mathrm{Max}}^{2}}{\mathrm{MSE}}\right),$$

where:

(21) $$\mathrm{MSE}=\frac{1}{MN}\sum_{i=1}^{M}\sum_{j=1}^{N}{\left[I(i,j)-K(i,j)\right]}^{2}.$$

The MSE calculation uses the common weight (*M*) and height (*N*) for both images and the pixel value in each position of the images (I(i,j) for the reconstruction and K(i,j) for the input). Then, the mean value is calculated. The inclusion of this additional metric enhances the reliability of the results [43].

The model was evaluated through successive tests using spectrogram segments collected under normal conditions, which constituted the majority of the data. Additionally, segments representing known abnormalities were tested to ensure that the model could distinguish between normal and abnormal conditions. This was achieved by evaluating the metrics SSIM and PSNR over time. Under normal conditions, the models were trained to minimize the level of reconstruction error, leading to high SSIM and PSNR values. However, when the model was exposed to abnormal data (distinct from the data used during training), the reconstruction quality may degrade, resulting in significantly lower SSIM and PSNR values.

To deal with abrupt oscillations of the metrics between segments, the Kalman filter was used [44] to smooth them. This filter was crucial for the data trend analysis, as it minimized the impacts of noise and irregular fluctuations, thereby providing a clearer representation of the underlying signal. By operating similarly to the moving average, a more effective and dynamic operation was ensured, providing a predictive background instead of the moving average.

### 4.4. Fault Classification

Following fault detection, the next step was to label the issue by reaching a robust diagnosis for the wind turbine’s status. For this, deep learning models known as specialist auto-encoders were developed. These had the following structures: Conditional Denoising Auto-Encoder (CDAE) and Conditional Variational Auto-Encoder (CVAE). Both of them are extended versions of DAE and VAE and were trained with spectrograms that represented specific known fault conditions.

**Figure 15:**
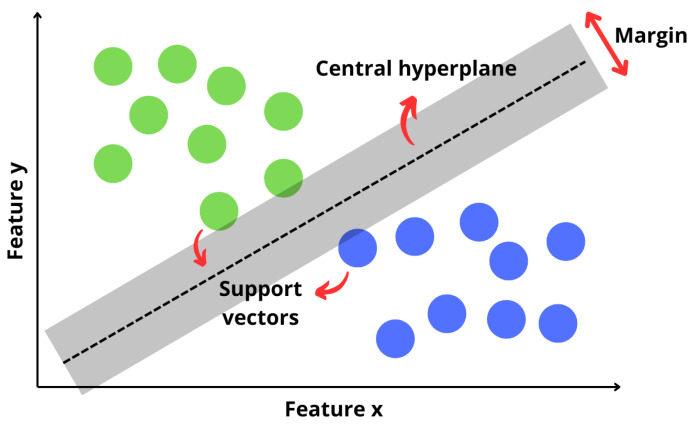
SVM separating the data linearly.

After data projection to the latent space of the CDAE and CVAE, the SVM algorithm, introduced by Vapnik [45], was used to classify the fault condition. This algorithm is commonly used for regression and classification, where, through a hyperplane, it classifies unknown data based on a previously labeled dataset during training. Figure 15 shows an example of how the data are represented in a bi-dimensional plane and the data grouping that the SVM performs:

The core idea of the SVM is to transform the input data into a higher-dimensional space using a kernel function and then create an Optimal Separating Hyperplane (OSH) to distinguish between the two classes in this transformed space. The data vectors close to the margin define and carry the information about the OSH and, instead of the model depending on all data, the support vectors define the threshold. Equation (22) carries out the binary classification task of the SVM,

(22) $$g\left(x\right)=\mathrm{sign}\left[\sum_{i=1}^{n}a_{i}y_{i}K\left(x_{i},x\right)+b\right],$$

where the decision function g(x) is described for *n* samples. It depends on the training coefficient $a_{i}$, the binary label $y_{i}$ (−1 or 1), and the kernel function $K(X_{i},i)$, which measure the similarity between the points added to the bias *b* of the neuron. The output of this function returns a value of −1 or 1 (this determines the region in which the data are labeled).

For the present model, the data projected in the latent space by the CDAE and CVAE had high dimensionality and could show a non-linear form during data clustering. The SVM carried out mapping in a superior dimension, allowing linear separation. The algorithm identified support vectors and used them to establish borders.

## 5. Experimental Results

Five wind turbines were monitored to produce the experimental results, and individual audio recorder hardware was developed and installed on each piece of equipment. The wind turbines were identified by individual ID codes: FAI2-07, FAI2-12, FAI1-05, FAI1-07, and FAI4-07.

The training process involved 20k spectrograms that were balanced in terms of wind speed and the five different wind turbines. These spectrograms were extracted from segments from the spectrogram images of three known healthy machines: FAI2-12, FAI1-05, and FAI1-07.

The grayscale color map was applied to the images in order to reduce the computational cost, as mentioned in the proposed method. The NFFT length of the spectrograms was set to 16,384 Hz to provide a balance between the resolution quality and processing.

To ensure the auto-encoder’s reliability, as shown in the following subsections, two inference processes were used to detect two known fault conditions in specific wind turbines.

### 5.1. Bearing Fault Detection

**Figure 16:**
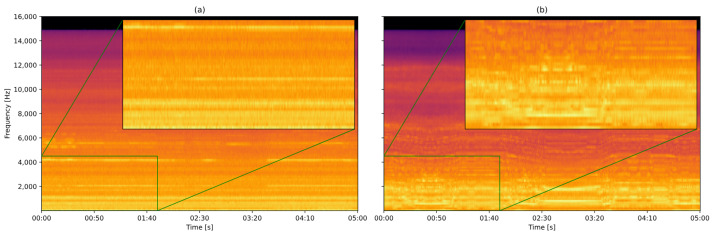
Comparison between a spectrogram of the sound produced by the wind turbine (**a**) FAI1-07 (desirable condition) and (**b**) FAI2-07 (bearing emitting vibrations) recorded at the same moment.

For this first inference to test the algorithm, segments of data representing normal conditions were captured from four wind turbines and one specific wind turbine (FAI2-07) with a known bearing fault that was detected by the local operators. This condition can be observed through a visual inspection of the spectrogram, as shown in Figure 16, where the excessive vibrations emitted by the bearing components are highlighted. These vibrations can have various causes such as material fatigue, inadequate lubrication, misalignment, excessive load, or improper installation [46]:

As long as the models have been trained with spectrograms (such as presented in Figure 16a), the reconstruction loss of Figure 16b will be higher, and, consequently, show low similarity.

**Figure 17:**
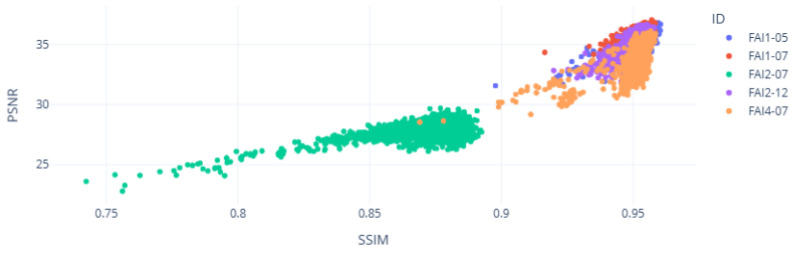
Inference of the VAE in the SSIM and PSNR analysis.

**Figure 18:**
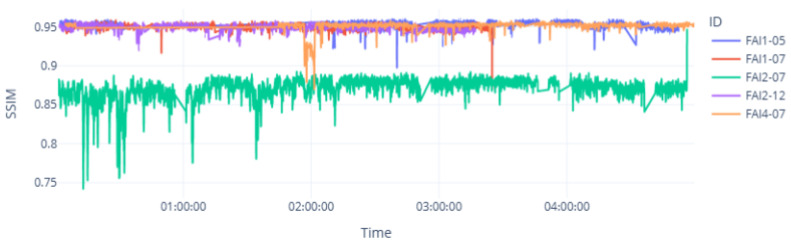
Inference of the VAE’s SSIM metric in the time domain.

**Figure 19:**
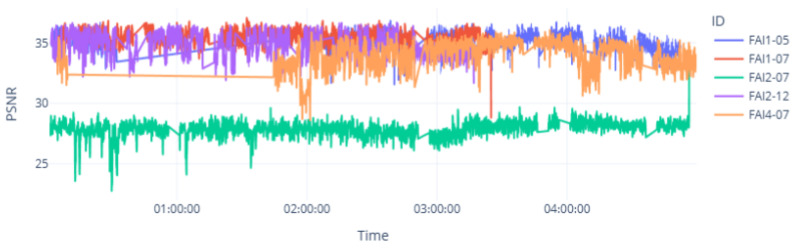
Inference of the VAE’s PSNR metric in the time domain.

Taking these aspects into account, Figure 17 presents an overview of the data grouping using the metrics collected for the five distinct wind turbines:

Each dot represents a spectrogram segment of 5 s from the audio recorded in each wind turbine. The metrics are represented on the axes and used to analyze the level of similarity between the output and input. It can be seen that the wind turbine FAI2-07 had lower levels of similarity for both metrics SSIM and PSNR than the other wind turbines. This turbine had a known bearing fault condition. The time-domain analysis shown in Figure 18 and Figure 19 shows the low level of similarity for this wind turbine throughout the time range captured.

The location of the blue line above the other lines indicates that the wind turbine FAI2-07 had a low level of similarity between the reconstructed and input unseen data.

Figure 19 also presents the PSNR for the same inference data, which provide the same results. It demonstrates a more sensitive metric that is capable of detecting subtle variations. These variations (often less evident in other metrics) can be critical for precisely identifying faulty conditions.

Based on the graphical analysis of each metric, Table 2 presents the statistical analysis for each individual wind turbine over this one-day analysis period:

The low similarity observed in the average FAI2-07 SSIM and PSNR with the high oscillation of the SSIM compared with other wind turbines indicates the known gearbox fault by the operator’s team.

### 5.2. DFIG Fault Detection

**Figure 20:**
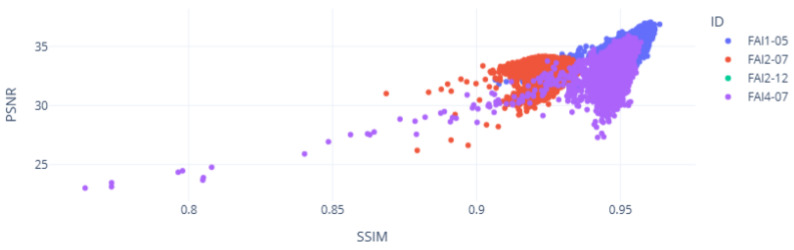
Inference of the VAE in the SSIM and PSNR DFIG analysis.

This second inference, conducted in November 2024, was detected by SCADA. This fault, which occurred in the wind turbine FAI4-07, is known as a DFIG inverter fault shutdown, which requires huge maintenance costs [47]. Figure 20 demonstrates the relationship between both SSIM and PSNR metrics:

As discussed in the previous subsection, the PSNR metric continued to highlight the low level of similarity between the reconstruction and input for the FAI2-07 case. Furthermore, wind turbine FAI4-07 also exhibited a low level of similarity when compared to FAI1-05 (normal condition), indicating successful fault detection.

Table 3 translates these results in statistical terms, ensuring detection from the auto-encoder:

In addition to the known fault in FAI2-07, the low level of similarity in the SSIM and especially in the PSNR average values can be observed by the standard deviation values.

### 5.3. Fault Classification

After successive inferences to test the model and define the metric threshold to distinguish healthy and non-healthy conditions, the segments were sent to the specialist AIs.

**Figure 21:**
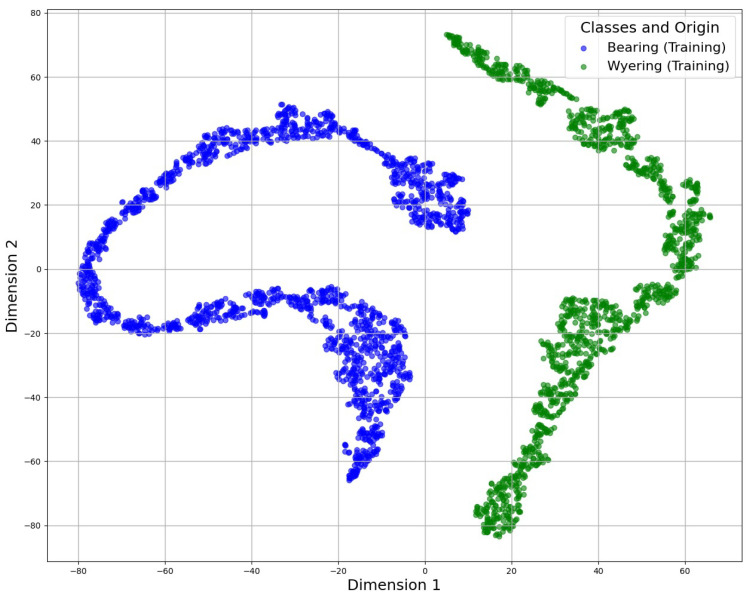
Latent space generated by the CDAE.

**Figure 22:**
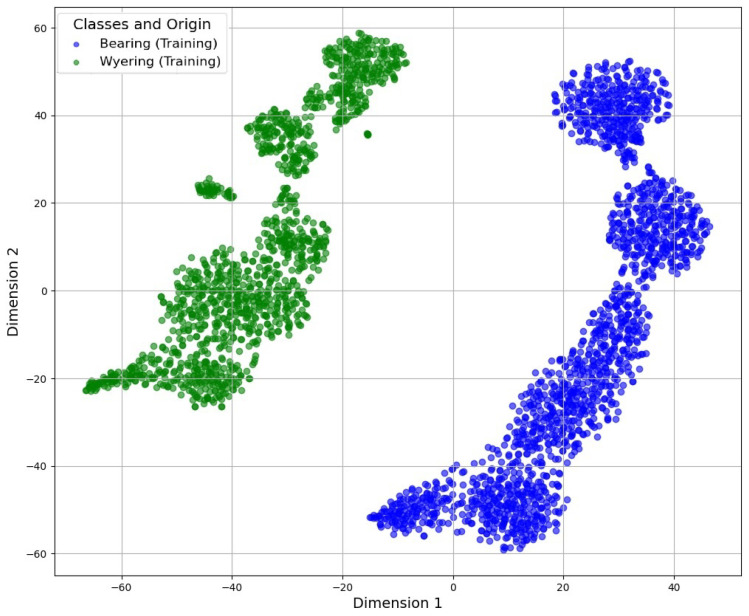
Latent space generated by the CVAE.

The specialist AIs, previously trained with the bearing fault spectrograms, received these unknown data with a low similarity level and classified them properly, as demonstrated in Figure 21 and Figure 22, which allowed the clustering of training data for the different conditions captured. This clustering used the t-distributed Stochastic Neighbor Embedding (t-SNE) algorithm to group similar data:

The CVAE approach created a latent space in which the input data were encoded. This latent space captured the essential characteristics of the spectrograms, enabling a compact and meaningful representation of the data. Similarly, the CDAE created a latent space and employed denoising techniques to enhance the reconstruction of the spectrograms, focusing on the removal of noise that could mask early fault signals.

The latent space shows that the known faults in the bearing and wye ring, which was also a failure identified by the operators, were clustered into two different groups. This feature ensured that the CDAE and CVAE could group and cluster different data represented by the segments of the spectrogram into a bi-dimensional space labeled as “Dimension 1” and “Dimension 2”, representing a generic depiction of the data variation.

**Figure 23:**
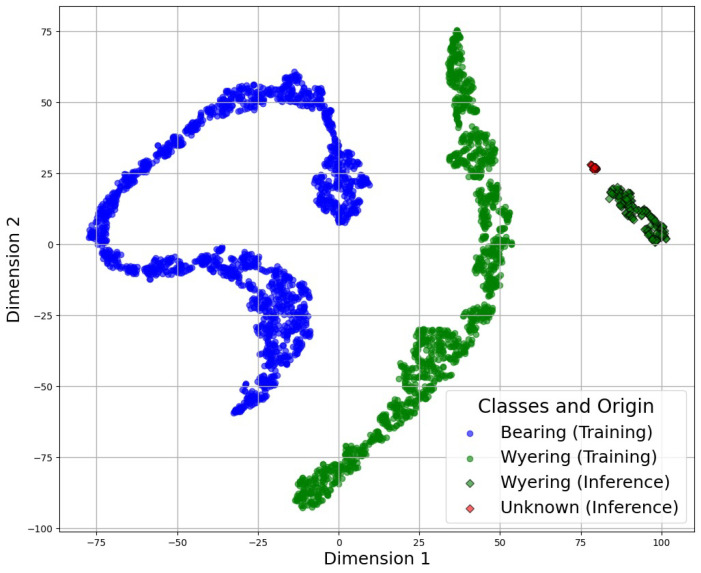
Application of the SVM in the CDAE latent space for wye-ring fault detection.

**Figure 24:**
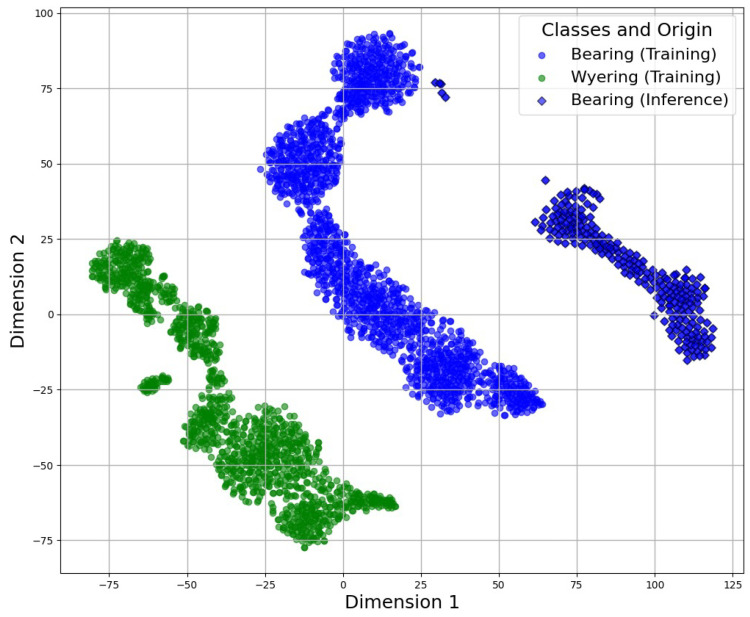
Application of the SVM in the CVAE latent space for bearing fault detection.

After this projection into the latent space, the SVM algorithm was applied to classify the different fault conditions. Figure 23 and Figure 24 demonstrate the combination of the classification using the SVM algorithm and the latent space generated by the auto-encoders. Those figures compare the training and inference data, demonstrating the approximation between them:

This analysis compared the training data (shown with circles) with the inference data (shown with dark-colored diamonds) to determine their level of similarity. The unknown data clustered near to the known data, validating the precision of the SVM technique.

The SVM algorithm classified the fault condition based on a predefined threshold, which was determined by the accuracy of the corresponding classification. This ensured that fault detection was both precise and reliable, aligning with the model’s performance metrics to minimize false positives and negatives. If the accuracy fell below the predefined threshold (even if the classifier initially attempted to assign a segment to a specific group), it would ultimately be classified as unknown, with the possibility of being analyzed and incorporated into a future dataset.

Table 4 shows the CVAE and CDAE results for 200 samples (spectrograms segments) of labeled faults as well as noisy and normal conditions. For noisy audio, Gaussian distributions of 0.1, 0.2, and 0.3 were applied. In this example, an accuracy threshold of 90% was adopted:

Both specialist auto-encoders were trained with the bearing and wye-ring faults, recognizing them with high precision. For other conditions, the model tried to group the trained clusters, but the model classified them as unknown faults with low precision.

## 6. Conclusions

This paper introduced a comprehensive solution that can be used to mitigate fault conditions in wind turbines by advancing predictive maintenance strategies within wind farms.

The proposed solution involved the development of a specialized hardware device that was designed to capture the unique sound profile of each wind turbine. This hardware enabled the transmission of the recorded audio data to a cloud-based service, where they were processed and analyzed by advanced AI neural networks. The system could accurately identify potential faults and anomalies by leveraging state-of-the-art machine learning techniques.

The importance of the AI methodology employed in processing the audio data was emphasized. The results highlighted the potential of this approach for use in fault detection, demonstrating that sound analysis was a powerful and underutilized tool in the wind energy sector.

By extracting valuable behavioral patterns from the audio signals, this methodology enhanced wind turbines’ overall reliability and efficiency, contributing to more sustainable and cost-effective wind energy production. Generalist auto-encoders could classify a fault condition based on the low level of similarity between the reconstructed and input data.

Through successive tests using a standardized spectrogram, it was possible to establish a threshold for both metrics to distinguish between healthy and unhealthy conditions. Specialist auto-encoders, which operated through supervised learning, achieved an accuracy level of approximately 98% when classifying fault conditions, demonstrating high precision when labeling wind turbine issues.

The promising outcomes underscore the significance of integrating sound analysis as a critical feature for advancing the predictive maintenance paradigm in renewable energy systems.

### Future Work

In future work, this fault detection methodology could be extended to other fields that involve rotating machines and other applications through sound event detection in equipment. The principles demonstrated in this study are not limited to wind turbines but can be adapted to various sectors, including manufacturing, automotive, aerospace, and energy production.

One of the notable advantages of this approach is the versatility of the developed hardware. The hardware architecture, designed for capturing and analyzing sound data, could be directly used in other domains with minimal or no modifications to its structure. This adaptability reduces the need for extensive redevelopment efforts, making the solution cost-effective and time-efficient for deployment across different applications. By leveraging this hardware and the associated AI-based sound analysis methodologies, industries can enhance their predictive maintenance strategies, improve equipment reliability, and reduce operational downtime.

It is important to highlight that this model faces scalability limitations, meaning that if additional wind turbines are added for supervision, the metrics of the new equipment may drop to values that could be mistakenly interpreted as anomalous conditions. This could be due to the individual characteristics of each wind turbine, such as noise occurring in the nacelle structure, which may not necessarily indicate an unhealthy condition. To mitigate this issue, an analysis of the new equipment under normal operating conditions with distinct spectrograms is required so that the data collected can be incorporated into the dataset. This would allow the model to learn a broader representation of normal conditions and improve its accuracy.

## Figures and Tables

**Table 1:** Wind-speed conditions for the balanced dataset.

Condition	Wind Speed Range
Weak wind	<5 m/s
Medium wind	5–8 m/s
Strong wind	>8 m/s

**Table 2:** Statistical analysis of the inference determined using the average and standard deviation values for the bearing fault detection.

WT Id Code	SSIM		PSNR	
	Avg.	Std. Dev.	Avg.	Std. Dev.
FAI1-05	0.954	0.004	35.20	0.66
FAI1-07	0.950	0.003	35.47	0.59
FAI2-07	0.871	0.014	27.90	0.63
FAI2-12	0.949	0.004	34.80	**0**.96
FAI4-07	0.952	0.006	34.02	1.13

**Table 3:** Statistical analysis from the inference achieved using the average and standard deviation values for DFIG fault detection.

WT Id Code	SSIM		PSNR	
	Avg.	Std. Dev.	Avg.	Std. Dev.
FAI1-05	0.954	0.005	35.274	0.781
FAI2-07	0.923	0.005	33.180	0.704
FAI2-12	0.951	0.004	34.277	0.510
FAI4-07	0.949	0.007	33.632	1.173

**Table 4:** Precision for labeled and unlabeled data in the CVAE and CDAE classifications.

Type	Accuracy %		Fault Classification
	CVAE	CDAE	
Bearing	99	99	Bearing
Wye ring	98	98	Wye ring
Noisy audio 1—0.1	35	30	Unknown fault
Noisy audio 1—0.2	28	25	Unknown fault
Noisy audio 1—0.3	20	18	Unknown fault
Noisy audio 2—0.1	33	29	Unknown fault
Noisy audio 2—0.2	27	24	Unknown fault
Noisy audio 2—0.3	19	16	Unknown fault
Normal audio 1	22	18	Unknown fault
Normal audio 2	17	14	Unknown fault
Normal audio 3	8	7	Unknown fault

## Data Availability

The data used to support the findings of this study are available from the corresponding author upon request.

## Abbreviations

The following abbreviations are used in this manuscript. While these abbreviations are generally written within the text, this table serves to clarify and summarize them in one place:

AI	Artificial Intelligence
CDAE	Conditional Denoising Auto-Encoder
CNN	Convolutional Neural Network
CVAE	Conditional Variational Auto-Encoder
DAE	Denoising Auto-Encoder
DFIG	Doubly Fed Induction Generator
KL	Kullback–Leibler
MSE	Mean Squared Error
NDITs	Non-Destructive Inspection Technologies
NFFT	Nonuniform Fast Fourier Transform
OSH	Optimal Separating Hyperplane
PCA	Principal Component Analysis
PSNR	Peak Signal-to-Noise Ratio
ReLU	Rectifier Linear Unit
SCADA	Supervision, Control, and Data Acquisition
SELU	Scaled Exponential Linear Unit
SSIM	Structural Similarity Index Measure
STFT	Short-Time Fourier Transform
SVM	Support Vector Machine
t-SNE	t-distributed Stochastic Neighbor Embedding
VAE	Variational Auto-Encoder
